# Polycomb-Derived Posttraumatic Stress Disorder (PTSD) Signals Diverge From Classical Aging Pathways and Converge on Neuroimmune Genes

**DOI:** 10.7759/cureus.112717

**Published:** 2026-07-15

**Authors:** Ngo Cheung

**Affiliations:** 1 Psychiatry, Cheung Ngo Medical Limited, Hong Kong, HKG

**Keywords:** aging, cd40, genage, h3k27me3, microglia, neuroinflammation, polycomb, ptsd, twas

## Abstract

Background

Posttraumatic stress disorder (PTSD) is a trauma- and stressor-related disorder characterized by persistent intrusion symptoms, avoidance, negative alterations in cognition and mood, and alterations in arousal and reactivity after exposure to a qualifying traumatic event. Symptoms must persist for more than one month and cause clinically significant distress or functional impairment under the Diagnostic and Statistical Manual of Mental Disorders, Fifth Edition, Text Revision (DSM-5-TR) criteria. Reported PTSD prevalence in older adults varies according to diagnostic criteria and sampling frame; nationally representative studies have reported lifetime prevalence estimates of approximately 4.5% under the DSM-IV criteria and higher probable DSM-5 estimates when functional impairment was not included. Aging-related gene-set annotations may therefore provide one way to investigate biological vulnerability without directly measuring aging phenotypes. This secondary gene-set analysis tested whether supplied Polycomb-derived aging modules were competitively enriched in PTSD transcriptome-wide association study (TWAS) signals and whether their strongest gene-level signals were distinct from the curated GenAge human aging comparator. The post-hoc analyses were exploratory and hypothesis-generating.

Methods

PTSD S-PrediXcan/TWAS summary files were analyzed using absolute meta-Z-score ranking. Competitive gene set enrichment analysis (GSEA) used 2,000 gene-label permutations, and over-representation analysis (ORA) examined genes with absolute Z-scores greater than 3. Leading-edge overlap, signed directionality, top-gene comparisons, manual functional categorization, and sensitivity analyses after removing GenAge-overlapping genes were performed as secondary analyses.

Results

At the set level, GenAge showed the highest normalized enrichment score (NES = 1.0971; permutation p = 0.124938), while Module B was the strongest Polycomb-derived module but did not exceed GenAge (NES = 1.0474; p = 0.206397; false discovery rate (FDR) = 0.825588). None of the Polycomb-derived modules reached significance after FDR correction, and their ORA results were non-significant. Exploratory analysis of Module B showed larger top-tail absolute TWAS Z-scores than GenAge among the available top-50 output (median |Z|, 4.7845 versus 3.8822; nominal Mann-Whitney U p = 0.000474). Immune/major histocompatibility complex (MHC)/co-stimulation genes had the most negative descriptive mean Z-score, but no leading-edge or top-gene sign test showed statistically significant directional skew. Module B shared little leading-edge or top-gene overlap with GenAge.

Conclusions

Primary analyses did not demonstrate robust competitive enrichment of Polycomb-derived aging modules in PTSD TWAS. The Module B findings are secondary and exploratory. They generate a testable hypothesis that selected immune/MHC, adhesion, and membrane-signaling genes may contribute to PTSD vulnerability through a focal neuroimmune-adhesion interface that is partly separable from broad cellular aging programs. This possibility requires independent replication, formal pathway testing, and functional validation before mechanistic or translational conclusions can be drawn.

## Introduction

Posttraumatic stress disorder (PTSD) is a clinically heterogeneous trauma- and stressor-related disorder. Under the Diagnostic and Statistical Manual of Mental Disorders, Fifth Edition, Text Revision (DSM-5-TR), diagnosis requires exposure to actual or threatened death, serious injury, or sexual violence, followed by symptoms from four domains: intrusion or re-experiencing, avoidance, negative alterations in cognition and mood, and alterations in arousal and reactivity. Symptoms must persist for more than one month and cause clinically significant distress or impairment [1]. Some individuals recover after trauma exposure, whereas others develop persistent symptoms involving intrusive memories, avoidance, hyperarousal, mood disturbance, sleep disruption, and impaired social or occupational functioning. This variability has made it difficult to define one dominant biological pathway for the disorder. Existing clinical approaches, including trauma-focused psychotherapies and pharmacological treatment, are helpful for many patients but leave a substantial subgroup with incomplete response or chronic symptoms. This therapeutic heterogeneity has encouraged a shift toward mechanism-based approaches that can identify biologically meaningful subgroups rather than treating PTSD as a single molecular entity.

PTSD is also relevant to later-life mental health. In a nationally representative United States sample of adults aged 60 years and older, the lifetime PTSD prevalence was 4.5% under the DSM-IV criteria, with an additional 5.5% meeting the criteria for partial PTSD [2]. A later analysis using probable DSM-5 criteria reported a prevalence of 9.5% in older adults when the functional impairment criterion was not included, a methodological feature that may have inflated the estimate [3]. These findings indicate that prevalence estimates vary substantially with diagnostic definitions and ascertainment methods, but that PTSD remains clinically relevant in older populations. Immune and inflammatory pathways have become central to this discussion. Meta-analytic evidence supports altered inflammatory markers in PTSD, although effects vary across studies and populations [4]. Blood transcriptome work has also shown that PTSD is associated with immune dysregulation across sex and trauma types, with shared signals involving cytokine, innate immune, and type I interferon pathways [5]. These findings align with broader work in stress-related disorders showing that immune signaling can influence neural circuits involved in mood, motivation, threat processing, and behavioral adaptation [6,7]. Immunoneuropsychiatry has therefore become a plausible framework for understanding why some stress-related syndromes persist despite conventional treatment [8].

Aging adds another layer to this problem. PTSD is not only a disorder of acute trauma response; it may also interact with biological aging, cellular stress, and brain vulnerability over time. Accelerated DNA methylation age has been associated with PTSD and neural integrity, suggesting that age-related molecular processes may modify PTSD biology rather than simply accompany chronological aging [9]. Aging brain transcriptome studies have also identified coordinated changes in inflammation, oxidative stress, mitochondrial function, calcium regulation, synaptic plasticity, myelin turnover, and cognitive impairment [10]. These data suggest that aging-related transcriptional programs may alter the way neural and immune cells respond to stress.

A transcriptome-wide association study (TWAS) integrates genome-wide association study (GWAS) summary statistics with expression quantitative trait locus prediction models to test whether genetically predicted gene expression is associated with a phenotype. S-PrediXcan extends this framework to summary-level GWAS data and can generate tissue-specific gene-level association statistics without requiring individual-level genotype and expression data [11]. Gene-set analysis of TWAS Z-scores can then test whether predefined biological groups contain an excess of large-magnitude gene-level associations. However, this approach evaluates overlap with annotated gene sets rather than directly measuring chromatin state, cellular aging, or disease-associated expression.

Microglia are especially relevant in this context. They respond to neuronal “on” and “off” signals, participate in synaptic remodeling, clear apoptotic cells, and can shift toward inflammatory phenotypes under conditions of injury, aging, or chronic stress [12-15]. Neuroinflammation is also recognized as a contributor to neurodegenerative vulnerability, and age-related inflammatory changes can negatively affect neuronal function [16,17]. Although PTSD is not a classical neurodegenerative disorder, these findings raise the possibility that aging-related neuroimmune states may alter the threshold for stress-related psychiatric symptoms or their persistence. The present analysis did not include cell-type-resolved data, microglia-specific TWAS, single-cell data, or direct measures of neuroinflammation; therefore, any microglial interpretation remains hypothetical.

Epigenetic regulation provides a plausible bridge between aging and stress vulnerability. PTSD has been linked to genetic, epigenetic, and transcriptomic variation, including immune-related methylation and expression profiles [18,19]. Neuronal chromatin landscapes are developmentally regulated and can vary between individuals, supporting the broader idea that histone-mark states may shape psychiatric risk through effects on gene expression programs [20]. The current study did not measure Polycomb occupancy, H3K27me3, chromatin accessibility, or other epigenomic features in PTSD. Instead, it evaluated supplied gene sets that had been derived from or annotated using prior Polycomb-related aging work.

The supplied Polycomb-derived modules were interpreted according to an upstream annotation/preprint source [21]. According to that source, the module annotations included adhesion- or protocadherin-related biology for Module A; synaptic scaffold, neurotransmission, signaling, and adhesion-related biology for Module B; and stress-related or H3K27me3-loss-associated biology for Module C. These descriptions were treated as source annotations rather than as independently validated biological properties of the modules in the present analysis. The supplied code further indicates that the A, B, and C collections were formed by dividing the imported gene-set entries into three contiguous, near-equal blocks according to their file order. Thus, the exact module membership and any cross-module gene overlap depend on the supplied file structure and were not independently re-derived here.

Most aging analyses in psychiatric genetics rely on curated aging resources, such as GenAge, which emphasize genes involved in longevity, DNA repair, oxidative stress, genomic stability, metabolism, and cellular stress resistance [22,23]. Such gene sets are useful but may miss narrower age-related vulnerabilities that arise in particular cell types or chromatin contexts. The central question of this study was therefore whether Polycomb-derived aging modules show evidence of PTSD TWAS enrichment and whether any signal is biologically distinct from a conventional aging comparator.

The prespecified primary objective was to compare the competitive enrichment of the supplied Polycomb-derived gene sets with the GenAge human aging comparator in PTSD TWAS summary statistics. Secondary objectives were to describe leading-edge overlap, directional patterns, sensitivity to GenAge overlap, and gene-level differences. The decision to focus the extended post-hoc analysis on Module B was data-driven because Module B showed the highest NES among the Polycomb-derived parts; this emphasis was not an independent prespecified hypothesis and is therefore interpreted as exploratory.

## Materials and methods

Study design and analysis period

This was a secondary, hypothesis-generating gene-set analysis of supplied summary-level PTSD S-PrediXcan/TWAS results. It was not a new GWAS, a new TWAS, a direct test of Polycomb-mediated regulation, or a study of aging phenotypes. The analysis evaluated whether supplied aging-related gene sets overlapped with the distribution of PTSD TWAS gene-level Z-scores.

The supplied notebook and execution materials did not preserve a calendar month or year for the reported run. The code header identified the pipeline as “Extended Pipeline v260430,” but this label was not accompanied by an execution date. Therefore, the exact analysis period cannot be stated without inference and is reported as not recorded.

Data sources and TWAS processing

The analysis used PTSD S-PrediXcan/TWAS summary statistics provided as gene-level Z-scores. The specific upstream PTSD cohort, GWAS source, and tissue model details were not encoded in the supplied summary files and therefore are not specified here. Input files were loaded from the PTSD TWAS result directory using a generalized parser that accepted comma-separated, tab-separated, text, and whitespace-delimited files. Gene symbols and Ensembl identifiers were harmonized when both were available. For genes represented across multiple tissue files, meta-Z scores were computed using Stouffer’s method across available tissue-level Z-scores.

The upstream PTSD GWAS/TWAS provenance required for exact replication was not available in the supplied files. Specifically, the materials did not identify the original cohort, total sample size, number of PTSD cases and controls, ancestry composition, included tissues, reference transcriptomic or expression quantitative trait locus panel, TWAS software release, or dataset date/version. The present analysis therefore used the supplied gene-level outputs as fixed input and did not attempt to reconstruct the upstream TWAS.

S-PrediXcan is a summary-statistics-based TWAS framework that estimates the association between genetically predicted gene expression and a phenotype using GWAS summary data and reference expression prediction models [11]. The present code did not perform the original S-PrediXcan calculations; it parsed and combined the supplied tissue-level gene-level results.

All gene symbols were converted to uppercase for matching. Ensembl aliases were mapped to gene symbols where possible, including version-stripped Ensembl identifiers. Mouse-derived module genes were analyzed as their supplied human gene-symbol counterparts. No additional undocumented orthology inference was performed during the reported run. This distinction is important because the reported results depend on the supplied gene-set definitions and the gene harmonization procedure used in the pipeline.

The available gene-set counts were as follows:

The GenAge Human set contained 307 unique input genes. Of these, 230 were detected after harmonization, while 77 were not detected in the supplied PTSD data.

The combined Polycomb-derived gene set contained 1,649 unique input genes. Of these, 1,168 were detected after harmonization, while 481 were not detected in the supplied PTSD data.

Module A contained 760 unique input genes. Of these, 553 were detected after harmonization, while 207 were not detected in the supplied PTSD data.

Module B contained 579 unique input genes. Of these, 429 were detected after harmonization, while 150 were not detected in the supplied PTSD data.

Module C contained 572 unique input genes. Of these, 384 were detected after harmonization, while 188 were not detected in the supplied PTSD data.

“Not detected” indicates that a supplied gene was not present in the final meta-Z dictionary after the available symbol/Ensembl matching procedure. The supplied execution output did not preserve separate counts for direct symbol matches, Ensembl-alias matches, duplicate rows removed, or identifiers rejected specifically because of formatting. These counts therefore should not be interpreted as pure identifier-error counts.

Cross-tissue Meta-Z calculation

For each gene, the code calculated an unweighted Stouffer meta-Z across the available tissue-level Z-scores using the formula:

meta-Z = (z1 + z2 + ... + zn) / square root of n

Each available tissue contributed one unweighted Z-score. No sample-size weights, inverse-variance weights, tissue-specific reliability weights, or eQTL prediction-performance weights were applied. Missing tissue values were omitted from the calculation, and no missing-value imputation was performed. The resulting meta-Z values were used for all primary ranking and post-hoc analyses.

Because cross-tissue Z-scores may be correlated through shared regulatory architecture, linkage disequilibrium, or shared prediction models, the unweighted Stouffer calculation should not be interpreted as a fully independent meta-analysis of tissues.

Gene sets and comparator

The focal gene sets were three supplied Polycomb-derived aging modules, labeled A, B, and C. The module annotations provided for interpretation described Module A as enriched for protocadherin or adhesion-related genes, Module B as enriched for synaptic scaffold, neurotransmission, signaling, and adhesion-related genes, and Module C as enriched for stress-related or H3K27me3-loss-associated genes according to the supplied upstream annotation source [21]. The analysis also included a combined “all” Polycomb-derived set. The comparator was the GenAge human gene set, a curated aging-related gene resource derived from the Human Ageing Genomic Resources [22,23].

The supplied code read GMT- and TXT-format files, sanitized set names, uppercased gene symbols, removed duplicate genes within each flattened collection, and preserved the order of the imported set entries. The keys were then divided into three contiguous near-equal blocks using a computational array split to form A, B, and C. The source materials did not provide a formal species-specific inclusion rule, repository release identifier, independent module-generation algorithm, or audit of genes occurring in more than one flattened module. Accordingly, A/B/C membership should be regarded as supplied computational collections rather than independently validated biological modules.

The GenAge file was supplied as GenAge_Human/genage_human.csv and contained 307 unique gene symbols before matching to the PTSD data. A release or build identifier was not included. The 307-gene count is compatible with later Human Ageing Genomic Resources descriptions of a human aging-related gene dataset containing more than 300 genes, but the exact GenAge build used here cannot be confirmed from the supplied files [22,23].

The number of genes detected in the PTSD TWAS data differed across sets. GenAge had 230 detected genes out of 307 supplied genes. The combined Polycomb-derived set had 1,168 detected genes out of 1,649. Module A had 553 detected genes out of 760, Module B had 429 detected genes out of 579, and Module C had 384 detected genes out of 572.

Gene set enrichment and over-representation analysis

The primary enrichment analysis ranked genes by absolute PTSD TWAS Z-score. This ranking was chosen to test whether a gene set was enriched for strong PTSD TWAS associations regardless of direction. The choice was therefore direction-agnostic and did not itself test a repression hypothesis. Signed directionality was evaluated only in secondary analyses.

Competitive GSEA used a weighted running-sum statistic with exponent 1.0; consequently, the running-sum hit contribution was proportional to the absolute meta-Z score. For each set, the observed enrichment score was compared with a competitive gene-label permutation null consisting of random gene sets of the same detected size. The reported run used 2,000 permutations and the default random seed of 42. The normalized enrichment score was calculated by dividing the observed enrichment score by the mean null enrichment score of the corresponding sign. The permutation null was cached by set size.

The reported normalized enrichment score, permutation p-value, and false discovery rate were computed using 2,000 permutations. Benjamini-Hochberg correction was applied across the four Polycomb-derived parts for the GSEA and ORA results. GenAge was treated as a benchmark comparator and was excluded from the Polycomb-specific FDR calculation.

A secondary over-representation analysis tested whether each gene set was enriched among genes with an absolute Z-score greater than 3. The background was the full ranked TWAS gene universe. Hypergeometric p-values were calculated for overlap with this high-association gene set. The threshold of an absolute Z-score greater than 3 was selected as an exploratory high-association cutoff rather than as a genome-wide significance threshold. The number of genes exceeding this threshold in the complete ranked universe was not retained in the supplied output.

For a gene set with K detected genes, a universe of M analyzed genes, n significant genes defined by an absolute Z-score greater than 3, and x overlapping genes, the ORA p-value was calculated as the hypergeometric upper-tail probability of observing x or more overlapping genes. The supplied ORA function returned overlap counts and p-values but did not calculate an odds ratio or other ORA effect size. Because the complete universe size and absolute Z-score greater than 3 count were not preserved in the supplied appendix, an ORA effect size was not reconstructed.

Post-hoc gene-level analyses

Because set-level enrichment can obscure strong signals concentrated in a subset of genes, secondary post-hoc analyses were conducted after inspection of the primary NES values, with Module B selected as the focal part because it had the highest Polycomb-derived NES. This selection was data-driven and may introduce selective emphasis. Leading-edge genes were extracted for each set. A leading-edge gene was defined as a detected set member occurring up to the maximum deviation of the weighted running-sum statistic for a positive enrichment score; for negative enrichment scores, the corresponding tail-side members were used in the post-hoc extraction function.

Directionality was assessed using signed PTSD TWAS meta-Z values, where negative Z-scores indicate negative association between genetically predicted expression and PTSD in the TWAS model. These signed values should not be interpreted as direct evidence of measured repression in all disease-relevant cells.

Several follow-up tests were performed. First, leading-edge overlap between each Polycomb-derived set and GenAge was quantified using overlap counts and Jaccard indices. Jaccard indices were treated as descriptive overlap measures; no inferential Jaccard threshold or formal significance test was prespecified.

Second, sign tests were used to evaluate whether leading-edge genes or top genes ranked by absolute Z-score showed a significant bias toward positive or negative Z-scores. The sign tests were two-sided exact binomial tests with a null probability of 0.50 for positive and negative signs. Zero-valued Z-scores were excluded from the positive/negative count.

Third, top contributing genes were ranked by absolute Z-score, and the top 20, 30, and 50 genes from Module B were compared with corresponding GenAge genes. These top-n cutoffs were exploratory post-hoc thresholds and were not prespecified before inspection of the set-level results.

Fourth, the top 50 Module B genes were manually categorized into functional groups. The supplied code assigned the first matching category using prefix or substring rules for immune/MHC/co-stimulation, adhesion/cytoskeletal, synaptic transmission, signaling hubs, guidance/transporters/other, and unclassified genes. The categorization was performed by the author alone. Ambiguous genes were assigned according to the first matching rule, and no independent expert adjudication was performed.

Formal Enrichr or g:Profiler enrichment was not available in the supplied post-hoc output, so these functional categories should be interpreted as descriptive rather than as formal pathway enrichment results. The optional gseapy/Enrichr code path did not produce a formal enrichment table in the supplied output.

Fifth, top-gene absolute Z-score distributions were compared between Module B and GenAge using a Mann-Whitney U test. The comparisons were two-sided. The supplied code called the SciPy Mann-Whitney U implementation without explicitly specifying an exact or asymptotic method; the p-values therefore follow the default behavior of the installed SciPy version, which was not recorded. The supplied top-tail output reported U statistics and p-values but did not systematically report rank-biserial correlations or Cliff’s delta.

Finally, each Polycomb-derived set was retested after removing genes that overlapped with GenAge to assess whether any signal persisted independently of shared aging genes. Genes were considered overlapping if their harmonized uppercase symbols were present in both the Polycomb-derived set and GenAge. The numbers removed were 61 genes from the combined set, 28 from Module A, 24 from Module B, and 31 from Module C. NES and permutation p-values were recalculated after removal using the same ranked PTSD list and 2,000-permutation framework.

Statistical analysis

Nominal statistical significance was defined as p < 0.05, and multiple-testing significance for the primary Polycomb comparisons was defined as BH-FDR < 0.05. The four Polycomb-derived sets were the family for the primary GSEA and ORA FDR calculations. GenAge was retained as a benchmark comparator and was not included in that family.

No global family-wise FDR correction was applied across all secondary and post-hoc tests in the reported head-to-head run. Thus, p-values from top-tail comparisons, sign tests, and distributional comparisons are nominal and exploratory. Leading-edge overlaps, Jaccard indices, manually defined functional categories, and the GenAge-overlap sensitivity results were treated primarily as descriptive analyses. The generic pipeline contained an optional family-wise FDR function, but it was not enabled for the reported head-to-head/post-hoc output.

The analysis was implemented in Python using pandas, NumPy, SciPy, Matplotlib, seaborn, statsmodels, and standard-library modules. Exact Python and package versions were not preserved in the supplied materials. The supplied code did not use R, clusterProfiler, fgsea, or another formal pathway-enrichment package for the reported primary results.

Ethics

This study used summary-level PTSD TWAS results and predefined gene-set resources. No new human participants or animal experiments were recruited, enrolled, or performed. Additional institutional ethics approval and participant consent were therefore not required.

Data and code availability

The analysis was based on supplied PTSD TWAS summary statistics and predefined GenAge and Polycomb-derived gene sets. Derived tables and the analysis code are available from the corresponding author upon reasonable request, subject to restrictions applying to the original data sources. The supplied materials did not include the complete upstream PTSD GWAS/TWAS provenance or a versioned software environment, which limits exact replication.

## Results

Overall module enrichment

The primary competitive analysis was negative. At the gene-set level, GenAge showed the strongest enrichment signal in PTSD TWAS (Table 1). GenAge had a normalized enrichment score of 1.0971 with a permutation p-value of 0.124938. Although this was the highest score among the tested sets, it did not reach conventional statistical significance in the competitive GSEA framework. GenAge also showed over-representation among genes with absolute Z-score greater than 3, with 51 overlapping genes and an ORA p-value of 0.03559 (Table 2). This ORA result was a benchmark observation and did not establish robust competitive enrichment of the aging gene set.

**Table 1:** Primary competitive gene set enrichment analysis results for GenAge and Polycomb-derived gene sets in PTSD TWAS Note. Genes were ranked by absolute PTSD TWAS meta-Z score. GSEA FDR was calculated across the four Polycomb-derived parts and excluded the GenAge benchmark comparator. ΔNES values are descriptive and do not represent formal statistical comparisons between NES values. NES: normalized enrichment score; GSEA: gene set enrichment analysis; FDR: false discovery rate

Trait	Set	Role	Detected / Input	NES	GSEA p	GSEA FDR	ΔNES vs GenAge	Statistical descriptor
PTSD	GenAge Human	Comparator	230 / 307	1.0971	0.124938	N/A (benchmark comparator)	0.0000	Benchmark comparator; non-significant competitive GSEA
PTSD	Module B	Part	429 / 579	1.0474	0.206397	0.825588	−0.0497	Did not exceed GenAge NES; non-significant
PTSD	Module A	Part	553 / 760	0.9998	0.499250	0.863568	−0.0973	Did not exceed GenAge NES; non-significant
PTSD	All Polycomb-derived	Part	1168 / 1649	0.9857	0.647676	0.863568	−0.1114	Did not exceed GenAge NES; non-significant
PTSD	Module C	Part	384 / 572	0.9157	0.905047	0.905047	−0.1814	Did not exceed GenAge NES; non-significant

**Table 2:** Over-representation analysis among genes with absolute PTSD TWAS Z-score greater than 3. ORA p-values were calculated using a hypergeometric upper-tail test. GenAge was treated as a benchmark comparator and was excluded from the Polycomb-specific FDR calculation. The threshold of |Z| > 3 was used as a secondary exploratory cutoff for high-association genes. The supplied ORA implementation did not calculate an odds ratio, and the complete universe size and number of genes exceeding |Z| > 3 were not retained in the supplied output; therefore, the effect-size column is reported as not calculated rather than reconstructed. ORA: over-representation analysis; MHC: major histocompatibility complex

Trait	Set	Detected / Input	Overlap with |Z| > 3	ORA p	ORA FDR	ORA odds ratio	Statistical descriptor
PTSD	GenAge Human	230 / 307	51	0.035590	N/A (benchmark comparator)	Not calculated in supplied run	Benchmark comparator
PTSD	Module B	429 / 579	74	0.550138	0.992356	Not calculated in supplied run	Non-significant
PTSD	Module A	553 / 760	93	0.657353	0.992356	Not calculated in supplied run	Non-significant
PTSD	All Polycomb-derived	1168 / 1649	181	0.966058	0.992356	Not calculated in supplied run	Non-significant
PTSD	Module C	384 / 572	50	0.992356	0.992356	Not calculated in supplied run	Non-significant

The Polycomb-derived modules did not show statistically robust competitive enrichment. Module B was the strongest Polycomb-derived part, with an NES of 1.0474, a permutation p-value of 0.206397, and an FDR of 0.825588. Module B therefore approached GenAge more closely than Modules A or C, but it did not outperform GenAge and was not statistically significant. Module A had an NES of 0.9998 with a permutation p-value of 0.49925 and an FDR of 0.863568. The combined Polycomb-derived set had an NES of 0.9857 with a p-value of 0.647676. Module C had the weakest signal, with an NES of 0.9157 and a p-value of 0.905047.

The ORA results followed a similar pattern in that none of the Polycomb-derived sets showed convincing enrichment among genes with an absolute Z-score greater than 3.

Module B had 74 overlapping genes but an ORA p-value of 0.550138. Module A had 93 overlapping genes with an ORA p-value of 0.657353. The combined set had 181 overlapping genes but an ORA p-value of 0.966058, and Module C had 50 overlapping genes with an ORA p-value of 0.992356. These results do not support strong Polycomb-derived set-wide enrichment in the high-association PTSD TWAS tail.

The comparison with GenAge was also informative when GenAge’s enrichment score was evaluated against random sets of the same sizes as the Polycomb-derived parts. The probability of a random set of Module B’s size reaching the GenAge enrichment score was 0.026. The corresponding values were 0.0115 for Module A, 0.0005 for the combined set, and 0.0395 for Module C. These values were descriptive size-matched null calculations and were not formal tests of the difference between GenAge and each Polycomb-derived set. They were not included in the primary FDR family.

Exploratory gene-level findings in Module B

The following results were obtained after the negative primary set-level analysis and should be interpreted as secondary, post-hoc observations (Tables 3, 4). Although Module B did not outperform GenAge as a whole, its highest-impact genes showed larger absolute PTSD TWAS effects than the top GenAge genes in the available top-tail comparison. Among the top 50 genes, the median absolute Z-score was 4.7845 for Module B compared with 3.8822 for GenAge. The maximum absolute Z-score was also higher in Module B than in GenAge, 14.7232 versus 10.7767. The supplied top-50 comparison produced a nominal Mann-Whitney U p-value of 0.000474, but no global correction across the post-hoc analyses was applied.

**Table 3:** Top 20 Module B genes by absolute PTSD TWAS Z-score TWAS rank refers to the gene’s position in the full analyzed PTSD TWAS gene universe after ranking genes by absolute meta-Z score. Descriptive categories were assigned manually for exploratory interpretation and do not represent formal Gene Ontology, Reactome, Kyoto Encyclopedia of Genes and Genomes, Enrichr, or g:Profiler enrichment results. MHC: major histocompatibility complex

Gene	Signed Z	|Z|	TWAS rank	Descriptive category
CD40	-14.7232	14.7232	3	Immune / co-stimulation
GNB2	-10.6876	10.6876	31	Signaling hub
SEMA3F	-10.2855	10.2855	39	Guidance / adhesion
SLC38A3	-10.0914	10.0914	44	Transporter
HLA-DMA	-9.1751	9.1751	62	MHC / antigen presentation
HLA-C	-8.0350	8.0350	120	MHC / antigen presentation
CTNNA1	7.3756	7.3756	189	Adhesion / cytoskeletal
CTNND1	-7.2385	7.2385	201	Adhesion / cytoskeletal
STX1A	-7.0090	7.0090	224	Synaptic transmission
EFNA5	6.9041	6.9041	243	Adhesion / guidance
WASF3	6.7446	6.7446	269	Cytoskeletal
RHOA	6.0327	6.0327	415	Cytoskeletal signaling
HLA-DOB	-5.9957	5.9957	423	MHC / antigen presentation
HLA-DMB	-5.7730	5.7730	474	MHC / antigen presentation
EP300	5.7572	5.7572	482	Chromatin / signaling
ATP6V1D	-5.7336	5.7336	492	Signaling / vesicular
ABLIM3	-5.4858	5.4858	555	Adhesion / cytoskeletal
NEGR1	5.4675	5.4675	566	Neuronal adhesion
SSH2	5.4600	5.4600	573	Cytoskeletal signaling
GRIA1	5.3502	5.3502	608	Synaptic transmission

**Table 4:** Exploratory top-tail absolute-Z-score comparison between Module B and GenAge The supplied output preserved a formal Mann–Whitney comparison for the top 50 genes but did not preserve corresponding U statistics and p-values for the top 20 and top 30 comparisons. The top-20 medians were calculated from the supplied top-20 gene lists; the top-30 values were not available. The rank-biserial effect size for the top-50 comparison was derived from the reported U statistic using the sample sizes of 50 and 50; its sign reflects the Module B–GenAge ordering. Top-n subsets were exploratory post-hoc comparisons, and no global FDR correction was applied across these tests.

Top n	Median |Z|, Module B	Median |Z|, GenAge	MWU U	Nominal MWU p	Rank-biserial effect size
20	6.8244	5.7261	Not retained in supplied output	Not retained in supplied output	Not available
30	Not retained in supplied output	Not retained in supplied output	Not retained in supplied output	Not retained in supplied output	Not available
50	4.7845	3.8822	1757.5	0.000474	-0.406, derived from the reported U statistic

The strongest Module B signal was CD40, with Z = −14.7232 and absolute Z = 14.7232, ranking third in the full PTSD TWAS ranked list (Table 3). Other high-impact Module B genes included GNB2 with Z = -10.6876, SEMA3F with Z = -10.2855, SLC38A3 with Z = -10.0914, HLA-DMA with Z = -9.1751, and HLA-C with Z = -8.0350. Additional prominent genes included CTNNA1, CTNND1, STX1A, EFNA5, WASF3, RHOA, HLA-DOB, HLA-DMB, EP300, ATP6V1D, ABLIM3, NEGR1, SSH2, and GRIA1. This list contains immune recognition genes, membrane and adhesion genes, signaling hubs, and selected synaptic genes, but it does not by itself establish a single coherent pathway.

Descriptive functional categorization

Functional categorization of the top 50 Module B genes clarified the observed mixture of signals (Table 5). The largest category was adhesion and cytoskeletal genes, with 15 genes. This group showed near-neutral directionality, with 53.3% negative Z-scores and a mean Z-score of +0.187. Guidance, transporter, and other genes included nine genes, with 44.4% negative Z-scores and a mean Z-score of -0.654. Signaling hubs also included nine genes, with 55.6% negative Z-scores and a mean Z-score of -1.222. Synaptic transmission genes included eight genes, with 62.5% negative Z-scores and a mean Z-score of −1.113.

**Table 5:** Descriptive functional categories of the top 50 Module B genes Categories were manually assigned for descriptive purposes and were not formal pathway-enrichment terms. Category-level means are descriptive only and may be unstable in small groups, particularly for the unclassified category with n = 1. The directional percentages do not represent formal category-level hypothesis tests. MHC: major histocompatibility complex

Functional category	Genes, n	Negative, %	Mean Z	Descriptive note
Adhesion / cytoskeletal	15	53.3	0.187	Most numerous category; directionally mixed
Guidance / transporter / other	9	44.4	-0.654	Mixed guidance and transport-related genes
Signaling hubs	9	55.6	-1.222	Mild negative tendency
Immune / MHC / co-stimulation	8	62.5	-3.916	Most negative descriptive mean; includes CD40 and HLA genes
Synaptic transmission	8	62.5	-1.113	Negative-leaning descriptive pattern
Unclassified	1	100.0	-3.804	Single-gene category; not interpretable as a class

The immune, MHC, and co-stimulation category showed the strongest negative descriptive mean. This category included 8 genes, 62.5% of which had negative Z-scores, with a mean Z-score of −3.916. Key genes in this group included CD40, HLA-DMA, HLA-C, HLA-DOB, and HLA-DMB. The magnitude of these genes is notable for prioritization, but the category was manually defined, small, and not tested using a formal pathway-enrichment method. In addition, HLA/MHC associations may reflect correlated regulation and linkage disequilibrium across the MHC region rather than independent evidence for antigen-presentation biology.

Directionality of leading-edge and top-tail genes

Directionality was not statistically decisive (Tables 6, 7). In the top 20 Module B genes, 12 were negative and eight were positive, corresponding to 60.0% negative genes and a mean Z-score of -2.5571, but the sign-test p-value was 0.503445. In the top 30 genes, the split was exactly balanced, with 15 positive and 15 negative genes and a sign-test p-value of 1.0. In the top 50 genes, 28 were negative and 22 were positive, corresponding to 56.0% negative genes and a mean Z-score of −1.1623, but the sign-test p-value was 0.479888. Thus, the negative immune/MHC mean and the mild negative top-tail trend were descriptive observations rather than evidence of coordinated directional repression.

**Table 6:** Directionality of leading-edge genes Leading-edge genes are the set members contributing up to the maximum running-sum deviation. No set showed statistically significant directional skew by the two-sided sign test. Sign-test p-values were nominal and were not globally adjusted across all secondary analyses.

Set	Leading-edge genes, n	Positive	Negative	Negative, %	Mean Z	Sign-test p
All Polycomb-derived genes	530	255	275	51.9	-0.1626	0.40922
Module A	261	122	139	53.3	-0.1474	0.32199
Module B	173	88	85	49.1	-0.1346	0.87920
Module C	173	87	86	49.7	-0.1017	1.00000
GenAge	65	38	27	41.5	0.8950	0.21454

**Table 7:** Directionality of exploratory top-|Z| genes in Module B and GenAge Top-n subsets were selected for exploratory post-hoc comparison and were not prespecified analytical strata. Because genes were selected by absolute Z-score, the signed mean and median Z values should not be interpreted as independent measures of set-wide directionality. No top-n sign test was statistically significant, and no global correction was applied across the top-n tests.

Set	Top n	Positive	Negative	Negative, %	Mean Z	Median Z	Sign-test p
Module B	20	8	12	60.0	-2.5571	-5.7533	0.503445
Module B	30	15	15	50.0	-1.1013	-0.2479	1.000000
Module B	50	22	28	56.0	-1.1623	-3.8721	0.479888
GenAge	20	12	8	40.0	1.8113	4.8458	0.503445
GenAge	30	18	12	40.0	1.4364	3.7298	0.361595
GenAge	50	30	20	40.0	1.1249	3.3106	0.202639

Distinction from broad aging biology

Module B and GenAge showed low leading-edge overlap (Table 8). At the leading-edge level, Module B had 173 leading-edge genes and GenAge had 65. Their overlap was eight genes, with a Jaccard index of 0.0348 (Table 8). The shared leading-edge genes were CDC42, CTNNB1, EP300, FGFR1, HRAS, PIK3CB, PTK2, and PTPN1. The other Polycomb-derived sets also showed low overlap with GenAge: the combined set shared 12 leading-edge genes with GenAge, Module A shared 9, and Module C shared 4. Jaccard indices ranged from 0.0171 to 0.0348. These descriptive overlap values are compatible with partial distinction between the supplied gene sets, but they do not constitute an inferential test of biological independence.

**Table 8:** Descriptive leading-edge overlap with GenAge Jaccard indices are descriptive measures of overlap and were not formally tested for significance. Leading-edge gene lists were extracted from the supplied ranked PTSD TWAS data. PTSD: posttraumatic stress disorder; TWAS: transcriptome-wide association study

Part	Part leading-edge genes	GenAge leading-edge genes	Overlap	Jaccard	Shared leading-edge genes
All Polycomb-derived	530	65	12	0.0206	AGTR1; CDC42; CTNNB1; EP300; FGFR1; GCLM; HRAS; HSPA1B; MTOR; PIK3CB; PTK2; PTPN1
Module A	261	65	9	0.0284	AGTR1; CDC42; FGFR1; GCLM; HRAS; HSPA1B; MTOR; PIK3CB; PTK2
Module B	173	65	8	0.0348	CDC42; CTNNB1; EP300; FGFR1; HRAS; PIK3CB; PTK2; PTPN1
Module C	173	65	4	0.0171	AGTR1; EP300; HRAS; PIK3CB

The same pattern was seen among the top contributing genes. In the top 20, top 30, and top 50 comparisons between Module B and GenAge, the only consistently shared gene was EP300. The Jaccard index was 0.0256 for the top 20 genes, 0.0169 for the top 30 genes, and 0.0101 for the top 50 genes. This minimal overlap suggests that the top-ranked Module B and GenAge signals were not identical, although overlap alone cannot establish distinct causal biology.

The biological contrast between the two sets was also evident from their highest-impact genes. GenAge’s leading drivers included GPX1, CISD2, SIRT3, NBN, CLOCK, TP53BP1, ATM, H2AFX, XRCC5, EP300, TPP2, PLAU, PML, HMGB2, XRCC6, ERCC3, TERF1, MED1, HELLS, and GRB2. Many of these genes point toward oxidative stress, mitochondrial biology, DNA repair, genome stability, chromatin regulation, or cellular stress pathways. By contrast, Module B’s strongest genes included immune co-stimulation, MHC biology, membrane signaling, cytoskeletal organization, adhesion, and selected synaptic genes. This contrast supports candidate prioritization but should not be interpreted as proof that Module B represents a separate aging mechanism.

Removing GenAge genes from the Polycomb-derived sets did not reveal an independent significant set-level signal. For Module B, the full retest NES was 1.0484 with p = 0.217891, and after removing 24 GenAge-overlapping genes, the score decreased slightly to 1.0369 with p = 0.283358. The change in NES was -0.0115. The combined set, Module A, and Module C also weakened slightly after GenAge-overlapping genes were removed. None remained significant after removal. These findings reinforce the distinction between strong individual gene-level signals and the absence of robust set-wide competitive enrichment.

Finally, full-set absolute Z-score distributions did not differ significantly from GenAge. The median absolute Z-score was 1.3470 for Module B compared with 1.3371 for GenAge, with Mann-Whitney p = 0.71248. The corresponding medians were 1.3735 for Module A, 1.3129 for the combined set, and 1.2453 for Module C, none of which differed significantly from GenAge. The global Module B distribution was therefore not stronger than GenAge; the observed difference was concentrated in the exploratory top tail. Table 9 shows the summary of flagged post-hoc findings.

**Table 9:** Summary of flagged secondary and post-hoc findings These observations were generated after the negative primary set-level analysis. They are hypothesis-generating and should not be interpreted as confirmatory evidence of a Polycomb-mediated PTSD pathway. PTSD: posttraumatic stress disorder

Analysis	Finding	Result	Descriptive interpretation
Top-gene overlap	Module B ∩ GenAge	EP300 was the only shared gene at top 20, top 30, and top 50	Minimal top-gene overlap is compatible with distinction between the two top-signal profiles
Category pattern	Immune / MHC / co-stimulation	n = 8; 62.5% negative; mean Z = −3.916	Descriptive negative-leaning immune/MHC pattern; not formally enriched or independently tested
Category pattern	Synaptic transmission	n = 8; 62.5% negative; mean Z = −1.113	Descriptive negative-leaning synaptic pattern
Effect-size comparison	Top 50 Module B versus GenAge	Median	Z
Directional sign tests	Module B and GenAge top genes	No sign-test p-value was significant	No evidence for coherent positive or negative directional skew
Functional enrichment	Top Module B genes	Formal enrichment output unavailable/not retained	Manual categories should be interpreted descriptively only

## Discussion

Mechanistic insights

The principal finding of this study is the absence of robust competitive enrichment for the Polycomb-derived aging modules in PTSD TWAS. GenAge had the highest set-level NES, and Module B was the strongest Polycomb-derived part, but neither the primary GSEA results nor the Polycomb-derived ORA results provided statistically convincing set-wide evidence. The more specific Module B observations therefore represent exploratory gene-level prioritization rather than validation of the primary hypothesis.

**Figure 1:**
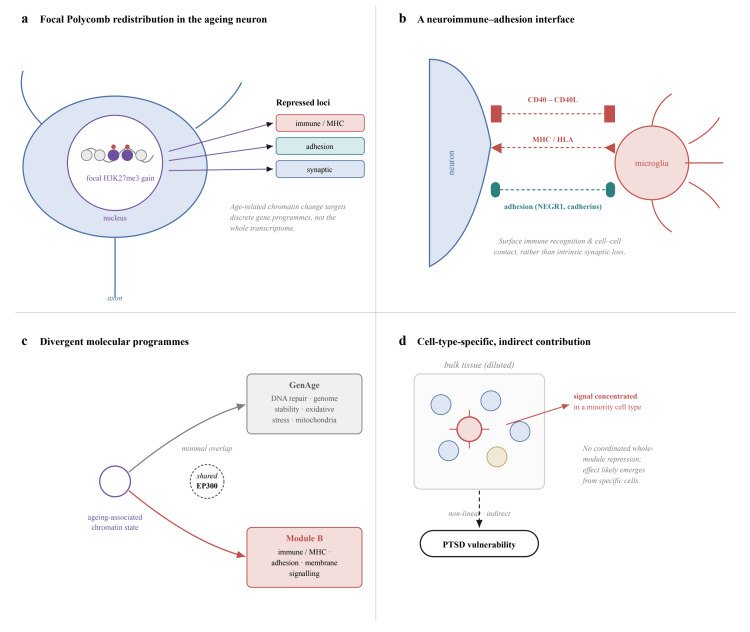
Exploratory schematic of a potential neuroimmune-adhesion pathway emerging from Module B genes in PTSD TWAS (a) Observed data: Module B was not significantly enriched at the set level, but selected genes in its top absolute-Z-score tail included CD40, HLA-DMA, HLA-C, HLA-DOB, HLA-DMB, SEMA3F, and adhesion- or membrane-related genes. (b) Interpretation: Module B showed low leading-edge and top-gene overlap with GenAge, while its full-set absolute-Z distribution did not differ significantly from GenAge. (c) Proposed biological model: these exploratory observations may point toward a potential immune-neuronal or neuroimmune-adhesion interface, but the model is speculative. (d) Important limitation: the current data do not resolve cell type, chromatin state, or causal mechanism. The Polycomb/H3K27me3 relationship is inferred from the upstream module source [17] and was not directly assessed in the PTSD TWAS data. The depicted glial or microglial engagement is hypothetical; bulk or unspecified TWAS results cannot establish cellular origin. The schematic is based primarily on exploratory Module B top-gene findings after non-significant module-level enrichment. PTSD: posttraumatic stress disorder; TWAS: transcriptome-wide association study Figure created by the author (Ngo Cheung) using Microsoft Powerpoint (Microsoft Corp., USA).

The more specific finding is that Module B contains a subset of high-impact genes with a biological profile that differs from the broader aging signature captured by GenAge (Figure 1). This distinction matters because set-level tests can miss concentrated biological signals when most genes in a module carry little association. In the present analysis, Module B’s overall NES was modest and non-significant, but its top genes included some of the strongest PTSD TWAS associations observed in the analysis. This pattern may indicate that a small subset of Module B genes warrants follow-up, but it does not demonstrate that the module as a whole is involved in PTSD.

The most plausible interpretation is that Module B may tag a neuroimmune-adhesion interface rather than a simple synaptic maintenance program. The original expectation for a Polycomb-related neuronal aging module might be that genes involved in neuronal identity, synaptic scaffolding, or neurotransmission would show coordinated evidence of repression or reduced expression-related association. That pattern was not clearly observed. Synaptic transmission genes did trend negative, but their mean Z-score was less extreme than that of the immune, MHC, and co-stimulatory group. Adhesion and cytoskeletal genes were numerous but directionally near neutral. The immune category, although smaller, showed the strongest negative mean Z-score and contained several of the most prominent genes.

These observations generate a narrower hypothesis that some PTSD-relevant Module B genes may lie at an immune-neuronal or cell-adhesion interface. However, the present data do not identify microglia, neurons, endothelial cells, peripheral immune cells, or any other specific cellular source. MHC genes and co-stimulatory molecules participate in immune recognition, antigen presentation, and cell-cell communication. In the brain, immune-related signaling can intersect with microglial activity, synaptic remodeling, and responses to injury or stress. Microglia are regulated by neuronal signals and can either support tissue homeostasis or contribute to inflammatory injury depending on context [12,16]. They also participate in clearance processes that can occur without overt inflammation under some conditions, while other states promote inflammatory activation [13,14]. The presence of CD40 and HLA genes therefore supports candidate prioritization for immune-neuronal studies, but it does not establish a CD40-mediated or antigen-presentation mechanism in PTSD.

This interpretation is consistent with broader PTSD and stress biology. Blood transcriptome data indicate that inflammatory and innate immune pathways are shared across different PTSD subgroups [5]. Transcriptomic work in the human brain has also emphasized that PTSD involves organized molecular changes rather than isolated single-gene effects [24]. In parallel, stress-related disorders have been linked to neuroimmune mechanisms in which peripheral and central immune signals influence neural plasticity and behavioral states [6,7]. The current findings do not prove that Module B genes act through microglia, but they are compatible with a model in which immune signaling and synaptic or adhesion biology may intersect.

The HLA/MHC findings require particular caution. The MHC region has extensive linkage disequilibrium, correlated regulation, and complex association structure. Consequently, strong TWAS associations for HLA-region genes do not by themselves establish independent causal effects or prove that antigen-presentation pathways are mechanistically altered in PTSD. Colocalization, fine-mapping, conditional analyses, and cell-type-resolved functional studies would be required before assigning a specific biological interpretation to these loci.

The contrast with GenAge further sharpens the exploratory interpretation. GenAge captured a broader cellular aging profile involving oxidative stress, DNA repair, mitochondrial function, and genomic maintenance. This is expected given the design of curated aging resources [22,23]. Module B, in contrast, highlighted immune recognition, membrane dynamics, cytoskeletal regulation, and adhesion. The minimal top-gene overlap, with EP300 as the only consistently shared gene among top 20, top 30, and top 50 comparisons, argues that the two top-tail profiles were not redundant. It does not, however, prove that Module B represents a distinct causal aging pathway. The current study measured overlap with predefined gene sets rather than aging biology itself.

At the same time, the lack of significant directional skew limits a simple repression model. If aging-associated H3K27me3 gain directly produced coordinated repression of PTSD-relevant genes, one might expect a stronger and statistically significant excess of negative TWAS Z-scores. That was not observed. Module B’s top 50 genes showed 56.0% negative Z-scores, and immune genes showed a stronger negative mean, but sign tests were not significant. The leading-edge directionality of Module B was even less supportive of coordinated repression, with 49.1% negative genes and a sign-test p-value of 0.8792.

The sign of a TWAS Z-score also requires caution. A negative Z-score does not directly mean that the gene is transcriptionally repressed in PTSD brain tissue or in aging neurons. It means that genetically predicted expression is negatively associated with PTSD under the model used. This relationship can differ by tissue, cell type, developmental stage, and regulatory context. Bulk TWAS can also dilute effects that are concentrated in microglia, inhibitory neurons, excitatory neurons, oligodendrocytes, or vascular-associated cells. Therefore, the most defensible mechanistic statement is that selected genes within a supplied Polycomb-derived collection may warrant investigation at immune-adhesion interfaces. The data do not demonstrate Polycomb repression, H3K27me3 redistribution, or coordinated cellular repression in PTSD.

Comparison with related PTSD, immunopsychiatry, and aging studies

The findings are broadly compatible with prior work showing that PTSD can involve inflammatory and immune-related molecular changes [4,5,24,25]. They also fit with immunopsychiatric models in which peripheral or central immune signals influence neural plasticity and behavioral states [6-8]. However, the present study differs from those studies because it did not directly measure inflammatory proteins, immune-cell activity, brain transcriptomic expression, or cell-type-specific molecular states. It therefore provides a gene-set prioritization analysis rather than an independent demonstration of neuroimmune activation.

The aging-related comparison also requires restraint. Accelerated DNA methylation age has been associated with PTSD and neural integrity [9], and aging brain transcriptome studies have identified alterations in mitochondrial, inflammatory, synaptic, and stress-related processes [10]. GenAge is designed to capture broad aging-related genes, whereas the supplied Polycomb-derived collections were based on narrower upstream annotations. The low overlap observed between Module B and GenAge may therefore reflect differences in gene-set construction as much as differences in biological mechanisms.

Strengths

This study has several strengths. First, it compares supplied Polycomb-derived collections with a curated aging comparator rather than interpreting one gene set in isolation. Second, it reports negative primary results rather than presenting the strongest post-hoc observations as the main enrichment finding. Third, it provides transparent gene counts, leading-edge overlap, sensitivity analyses, directionality tests, and top-gene lists. Fourth, it identifies a limited set of candidate genes, including CD40 and several HLA-region genes, that can be tested in independent datasets and experimental systems. Finally, the explicit distinction between set-level null findings and exploratory gene-level observations provides a framework for hypothesis generation without treating the post-hoc pattern as pathway validation.

Translational implications and clinical relevance

The translational value of these results lies in prioritization, not immediate clinical action. Module B did not show significant competitive enrichment, and its directional pattern was not statistically coherent. Nevertheless, the top-gene results provide a smaller candidate list than the full module.

The first potential implication is neuroimmune target prioritization. CD40 is particularly interesting because it is a co-stimulatory immune signaling gene rather than a conventional synaptic gene. HLA genes similarly point toward antigen presentation and immune recognition. These observations may justify testing whether CD40- or HLA-related regulatory variation is associated with PTSD-relevant phenotypes, but they do not justify immune-targeted treatment recommendations. Prior concepts in immunopsychiatry emphasize that immune-targeted approaches are most likely to work when patients are biologically stratified [7,8]. If the Module B pattern is independently validated, it could potentially inform such stratification research.

The second implication is aging-subtype stratification. PTSD research often discusses accelerated aging as though it were a single process. The present findings suggest that GenAge-like and Module B-like gene-set profiles may capture different aspects of aging-related annotation. GenAge-like biology may reflect cellular stress resilience, oxidative defense, DNA repair, and genome maintenance. Module B-like biology may instead reflect immune recognition, adhesion, membrane signaling, and neuron-glia communication. This distinction is a hypothesis about gene-set profiles, not evidence for biologically defined aging subtypes in patients.

The third implication is biomarker development. Blood transcriptomic work has already shown that PTSD is associated with immune pathway perturbation [5]. A refined Module B immune-adhesion signature could be tested against peripheral blood expression, methylation, proteomic markers, or brain-relevant cell models. The goal would not be to use the full Module B gene list as a diagnostic tool. Rather, the goal would be to determine whether a small, independently replicated group of immune and adhesion genes identifies a reproducible biological subgroup.

A practical roadmap follows from the data. In the short term, CD40, HLA-DMA, HLA-C, HLA-DOB, HLA-DMB, SEMA3F, and selected adhesion-related genes should be tested in independent PTSD TWAS datasets and in datasets with brain-region- or cell-type-resolved expression prediction. Human induced pluripotent stem cell-derived neurons, microglia, and neuron-microglia co-cultures may provide useful experimental systems, particularly when combined with stress-hormone exposure, inflammatory stimulation, or aging-like cellular stress. These experiments should be designed to distinguish immune-cell, glial, neuronal, endothelial, and peripheral-cell sources rather than assuming a microglial origin.

In the medium term, formal Gene Ontology, Reactome, Kyoto Encyclopedia of Genes and Genomes, g:Profiler, Enrichr, or comparable pathway analyses should be performed with an explicitly defined background and corrected for multiple testing. Orthogonal molecular studies should integrate DNA methylation, chromatin accessibility, histone-mark profiles, and expression data in PTSD-relevant tissues. In parallel, the immune-adhesion component of Module B should be tested as a stratification signature in independent PTSD datasets and compared directly with GenAge-like signatures.

In the longer term, carefully designed drug-repurposing or pathway-screening studies could examine CD40-related or MHC-related signaling in stress and neuroimmune models. Such work should remain exploratory and should not assume that pharmacological inhibition or stimulation of these pathways would be beneficial.

This translational framing also addresses a recurring problem in psychiatric genetics. Genome-wide and transcriptome-wide studies often identify statistically strong genes, but these genes can be difficult to organize into actionable biology. Here, the set-level result was underwhelming, but the gene-level pattern suggested a narrower hypothesis: PTSD vulnerability related to aging-associated gene-set annotations may involve immune-neuronal communication rather than only neuronal-intrinsic synaptic decline. This hypothesis is narrow enough to test, but broad enough to integrate with existing evidence for inflammation and immune dysregulation in PTSD [4,25].

Limitations

Several limitations should temper interpretation. First, the primary module-level results were weak and non-significant. Module B was the strongest Polycomb-derived module, but its GSEA p-value was 0.206397, and it did not outperform GenAge. The central hypothesis of robust Polycomb-derived module enrichment was therefore not supported. The post-hoc findings are hypothesis-generating and should not be presented as evidence that Module B is a validated PTSD pathway.

Second, the module definitions were supplied as input gene sets. The present analysis did not re-derive Polycomb modules from raw chromatin data. The biological labels for Modules A, B, and C were therefore treated as annotations rather than independently validated classifications. The supplied code formed A/B/C collections by dividing imported gene-set entries according to file order, and the source materials did not preserve a complete module-generation audit. This creates uncertainty about the stability and biological meaning of the module boundaries.

Third, the study did not directly measure aging. There were no age-stratified PTSD analyses, methylation-age measures, transcriptomic-age measures, or direct brain-aging phenotypes. The analysis tested overlap between PTSD TWAS signals and predefined aging-related gene sets; it did not demonstrate that aging mechanisms drive PTSD biology.

Fourth, the decision to prioritize Module B was data-driven. Module B was selected for extended post-hoc analysis because it had the highest Polycomb-derived NES. Although this is transparent, it creates a risk of selective emphasis. Parallel analyses of Modules A and C were not preserved at the same top-gene depth, so the apparent specificity of Module B should be interpreted cautiously.

Fifth, the upstream PTSD TWAS provenance was incomplete. The supplied materials did not identify the original GWAS cohort, sample size, case-control balance, ancestry, tissue prediction models, reference transcriptomic panel, software version, or dataset release date. This is a major reproducibility limitation because these features affect tissue interpretation, statistical power, linkage disequilibrium structure, and generalizability.

Sixth, mouse-to-human translation remains uncertain. The Polycomb-derived modules were based on aging-related biology from mouse neuronal contexts, whereas the PTSD TWAS data are human genetic association results. Even with careful gene-symbol harmonization, orthology and species differences can affect interpretation. In addition, chromatin marks, genetically predicted expression, and disease-associated expression are related but not equivalent layers of biology.

Seventh, TWAS results are sensitive to tissue models, eQTL architecture, linkage disequilibrium, and gene co-regulation. A strong TWAS Z-score does not identify a causal gene by itself. It also does not specify the disease-relevant cell type. This limitation is particularly important for HLA/MHC genes, where extensive linkage disequilibrium and co-regulation can produce correlated gene-level signals that are difficult to separate.

Eighth, functional interpretation was based on manual categorization rather than formal enrichment. Formal Enrichr, g:Profiler, Gene Ontology, Reactome, or Kyoto Encyclopedia of Genes and Genomes results were not available in the supplied output. The category labels were assigned by one author using keyword rules, without independent expert review, and may be unstable for multifunctional genes such as EP300, RHOA, CTNNA1, and HLA-region genes.

Ninth, directionality was not statistically confirmed. The immune/MHC category within Module B had a strong negative mean Z-score, and top Module B genes showed a mild negative trend, but sign tests did not reach significance. This weakens any model in which Polycomb-mediated repression directly and uniformly increases PTSD risk through coordinated downregulation of Module B genes. No direct conclusion about H3K27me3-mediated repression can be drawn from the present TWAS data.

Tenth, no cell-type deconvolution, single-cell sequencing, spatial transcriptomics, or microglia-specific TWAS was performed. The neuroimmune-adhesion and microglial interpretations are therefore speculative. Signals may arise from neurons, glia, endothelial cells, peripheral immune cells, or other cellular sources, and bulk or unspecified tissue models may dilute or combine these effects.

Finally, the analysis used 2,000 permutations. This is adequate for exploratory work but should be increased in a final confirmatory analysis. The post-hoc analyses also lacked a single global multiple-testing correction across all sign tests, top-tail comparisons, overlap summaries, and distributional tests. Independent PTSD TWAS datasets, formal pathway analysis, brain-region- or cell-type-specific models, and functional validation will be necessary before these findings can be translated into biomarkers or interventions.

## Conclusions

Primary analyses did not demonstrate robust competitive enrichment of Polycomb-derived aging modules in PTSD TWAS. GenAge remained the stronger set-level comparator, and Module B, although the strongest Polycomb-derived module, was non-significant. Exploratory examination of Module B identified high-impact genes at immune/MHC, adhesion, and membrane-signaling interfaces that were largely distinct from GenAge. These findings generate a testable hypothesis that focal neuroimmune-adhesion mechanisms may contribute to PTSD vulnerability separately from broad cellular aging programs. Validation in independent datasets and experimental systems is required before any mechanistic or translational inference.
